# Time-dependent effect of desensitization with wasp venom on selected parameters of the immune system

**DOI:** 10.1038/s41598-022-11155-2

**Published:** 2022-05-03

**Authors:** Łukasz Szymański, Weronika Urbańska, Martyna Ciepielak, Aleksandra Cios, Wanda Stankiewicz, Marta Stelmasiak, Agnieszka Rzeszotarska, Jolanta Korsak, Sławomir Lewicki, Andrzej Chciałowski

**Affiliations:** 1 Department of Molecular Biology, Institute of Genetics and Animal Biotechnology, Polish Academy of Science, Postępu 36A, Jastrzębiec, 05-552 Magdalenka, Poland; 2 Department of Infectious Diseases and Allergology, Military Institute of Medicine, Szaserów 128, 04-141 Warsaw, Poland; 3 Department of Microwave Safety, Military Institute of Hygiene and Epidemiology, Kozielska 4, 01-163 Warsaw, Poland; 4 Kazimierz Pulaski University of Technology and Humanities, Faculty of Medical Sciences and Health Sciences, 26-600 Radom, Poland; 5 Department of Clinical Transfusiology, Military Institute of Medicine, Szaserów 128, 04-141 Warsaw, Poland

**Keywords:** Immunotherapy, Immunization, Lymphocytes

## Abstract

The emergence of tolerance during Hymenoptera venom immunotherapy (VIT) is a complex process. The main goal of VIT is to induce a change from proinflammatory Th2 response to the Th1 response. However, the immune mechanism of acquiring rapid tolerance during VIT has not yet been fully understood. Therefore, we have analyzed (in 4-time points: 0, 2, 6, and 24 weeks after the initiation phase of VIT) the concentration of complement C3, C4, and C5 components, lymphocyte subpopulations (flow cytometry), as well as histamine and tryptase serum concentrations of 43 patients with wasp venom allergy (III and IV Müller grade) classified to ultra-rush treatment and 18 volunteers as the control group (CG). We observed that VIT affected the immune system by inducing changes in the complement system (decreased C3 and C4 compartment protein concentrations) and "normalized" the percentage of lymphocytes and neutrophils in the peripheral blood. Moreover, a significant increase in the percentage of nTreg in the blood of patients treated with VIT was observed. On the other hand, there were no changes in histamine or tryptase concentrations in the blood. Increased percentage of nTreg cells is a well-known mechanism by which VIT affects the immune system. Finally, VIT also modulated the concentrations of the complement components, which may be a previously unknown VIT mechanism of action.

## Introduction

Hymenoptera is an order of insects whose stings can cause an allergic reaction mediated by immunoglobulin E. The most common reactions (up to 26%) are edema, erythema, pain, and witness. However, a systemic reaction develops in 0.3–7.5% of the adult population of Europe and up to 32% of beekeepers^1^. Usually, the systemic reactions include skin, gastrointestinal, respiratory, and cardiovascular symptoms, which may develop separately or in combination. Anaphylaxis to an insect sting can cause terrifyingly rapid death, with initial cardiorespiratory arrest within 5–10 min of the venom sting exposition^2^. Therefore, venom-specific immunotherapy (popularly known as desensitization), consistsof the subcutaneous administration of increasing doses of the allergen (induction phase) to achieve maintenance dose tolerance, followed by the administration of maintenance doses. The induction phase, depending on the type of protocol, may last from three to five hours (ultra-rush, ultra-fast immunotherapy), several days (rush, rapid immunotherapy), or several weeks (conventional immunotherapy). Currently, this therapy is the most effective method of treatment^3^. Peripheral T cell tolerance to allergens is caused mainly by the generation of allergen-specific Treg cells and a decrease in Th2 and Th1 lymphocytes, initiated by IL-10 and TGF-β^4^. Subsets of Treg cells with distinct phenotypes and mechanisms of action include the naturally occurring, thymic selected CD4+ CD25+ Treg cells, and other cells. Natural regulatory T cells (nTreg) develop in the thymus. The cells are necessary for induction toleration of self-antigen. Decreased number of the cells in the blood or disturbances in their function is associated with a higher likelihood of autoimmune diseases and allergies. Moreover, Treg cells that occur in the blood may develop from the population of conventional CD4+ T cells, and these cells are called iTreg (induce T regulatory cells). iTreg may secrete IL-10 and TGF-beta, typical cytokines that attenuate immune cell response and increase self-antigens toleration^5,6^.

Here, we evaluate the short- (2 and 6 weeks) and long-time (24 weeks) effect of desensitization with wasp venom on selected immune system parameters in the blood. We focused on the percentage of WBC cells, lymphocyte phenotypes, and nTreg population, the level of selected complement components (C3, C4, and C5), and the concentrations of histamine and tryptase in the peripheral blood. The research was performed on the experimental group: patients with wasp allergy III/IV Müeller grade undergoing VIT treatment (to find an effect of VIT) and the control group: patients with wasp allergy I/II Müeller grade (to find potential differences between I/II and III/IV patients).

## Results

### IgE concentration

**Figure 1:**
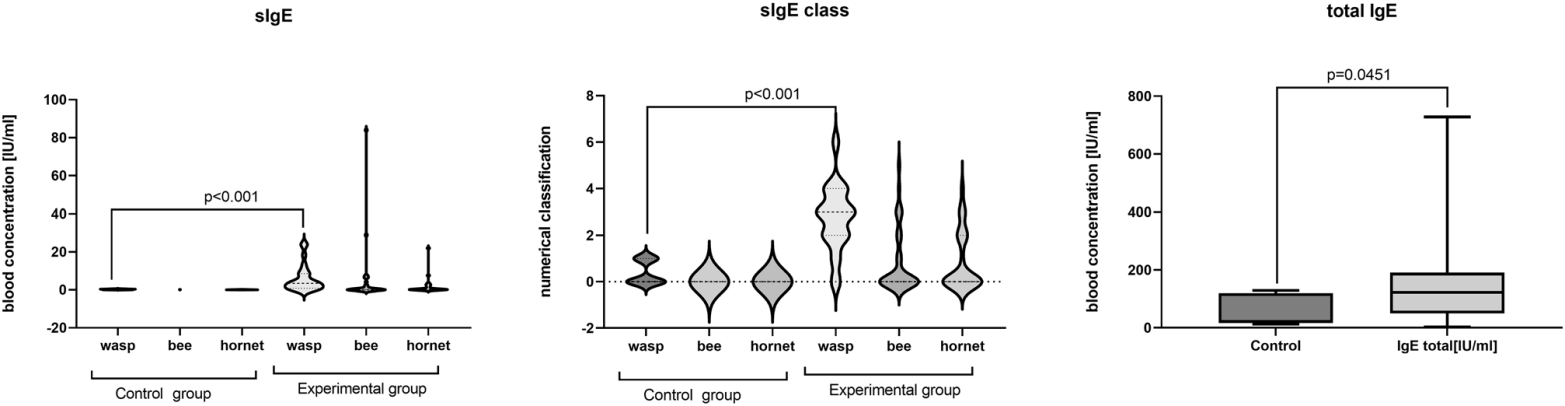
The total IgE and specific IgE wasp, bee, and hornet serum concentrations. The samples were collected at day 0 (control and examined group). *p* level of significance.

The primary qualification to the control or experimental group was performed according to Müeller criteria^7^. We chose patients with allergy symptoms after a wasp sting, however, we also evaluated total IgE and specific IgE (sIgE) for wasp, bee, and hornets venoms. Patients from the control group had several times lower concentrations of sIgE for wasp, which was associated with lower Müeller grade (one or below). Moreover, patients from the control group had lower total IgE concentrations in the serum than those from the experimental group. The results are presented in Fig. 1.

### White blood cells distribution

**Figure 2:**
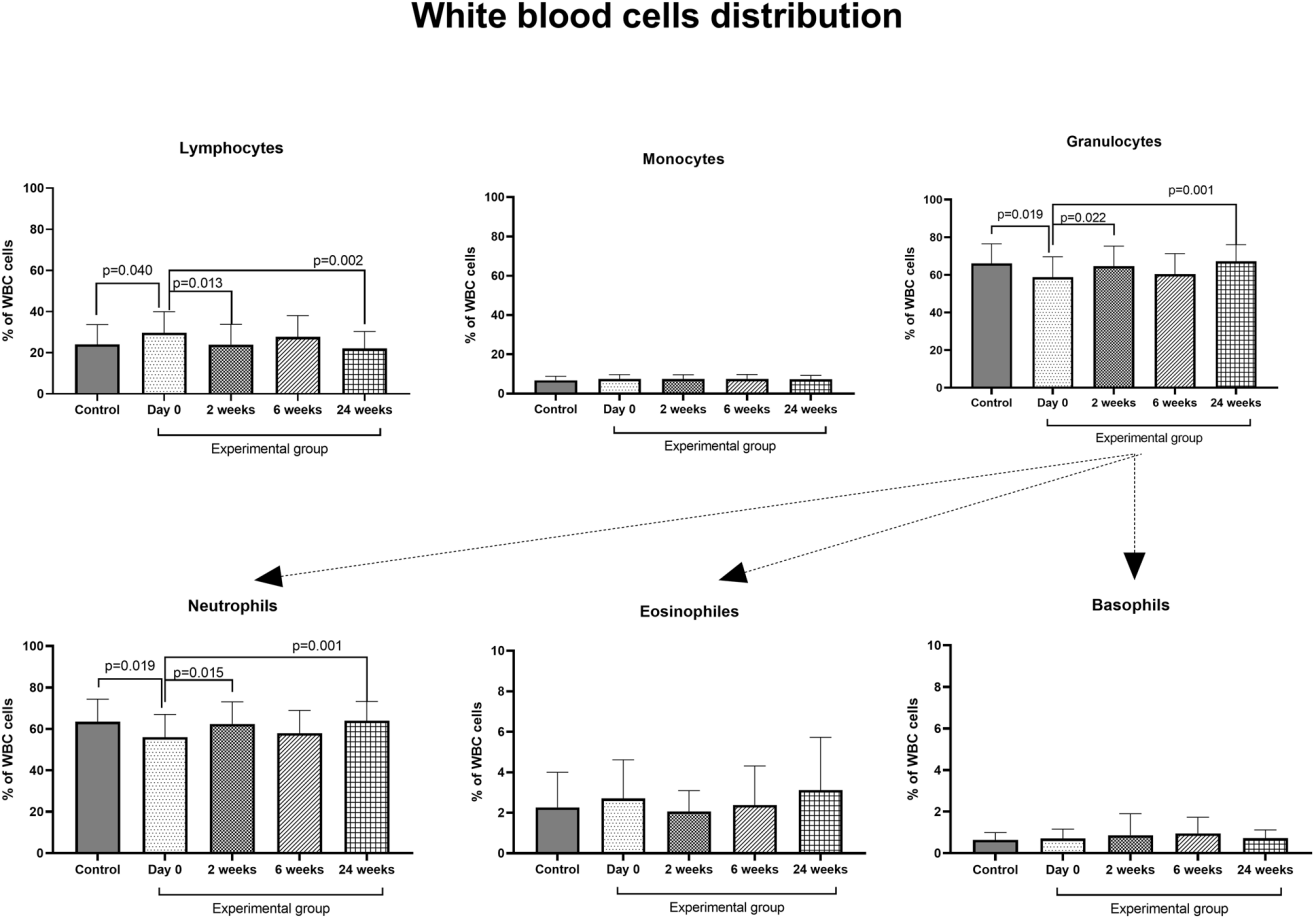
The effect of desensitization with Hymenoptera venom on white blood cells distribution in the blood. Blood was collected at day 0 (control and examined group), and 2, 6, and 24 weeks after the initiation phase of VIT (experimental group only). Results are presented as mean percentage ± SD. *p* level of significance.

The percentage of lymphocytes, monocytes, and granulocytes in 'patients' blood from the control group appropriately amounted to 24.01 ± 9.62%, 6.72 ± 2.07%, and 66.09 ± 10.45%. Patients from the experimental group (day 0) had a significantly higher percentage of lymphocytes (about 23%, p = 0.040, 29.63 ± 10.18%) and a lower percentage of granulocytes (about 22%, p = 0.019, 58.78 ± 10.67%). The lower percentage of granulocytes observed in the experimental group was associated with a lower percentage of neutrophils (about 22%, p = 0.019, 55.97 ± 10.83%). We did not observe significant differences in the percentage of monocytes, eosinophils, and basophils between the control and experimental groups. Desensitization of patients caused significant changes in both short (2 weeks) and long-time periods (24 weeks) after the initiation phase of VIT. The percentage of lymphocytes decreased (2 weeks—23.96 ± 9.68%; 24 weeks—22.02 ± 8.19%), and granulocytes (mainly neutrophils) increased (2 weeks—64.61 ± 10.55%; 24 weeks—67.16 ± 8.77%) to values observed in the control group. The results are presented in Fig. 2.

### Phenotype of lymphocytes

**Figure 3:**
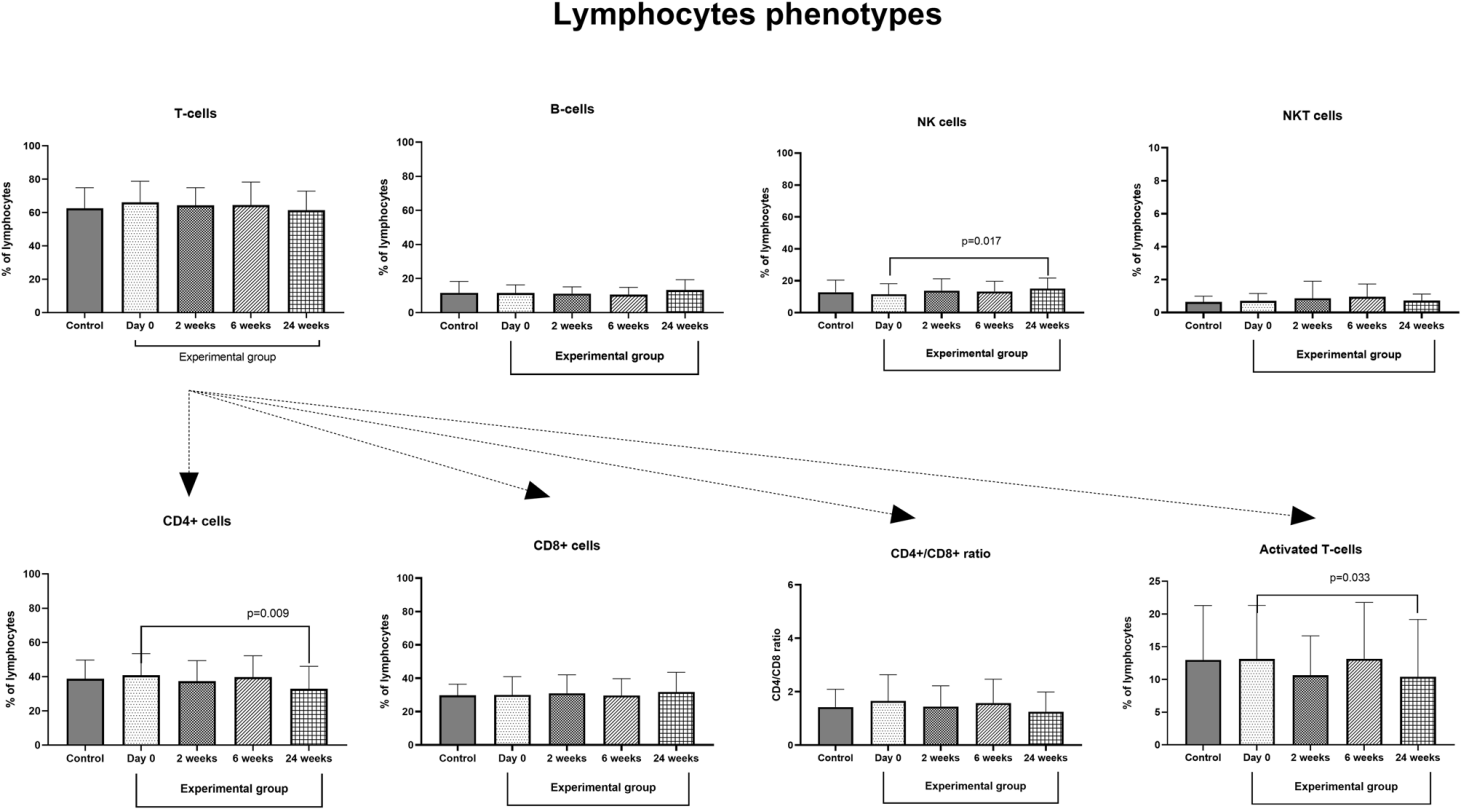
The effect of desensitization with Hymenoptera venom on populations of lymphocytes in the blood. Blood was collected at day 0 (control and examined group), and 2, 6, and 24 weeks after the initiation phase of VIT (experimental group only). Results are presented as mean percentage ± SD. *p* level of significance.

In the control group percentage distribution of lymphocytes were 62.47 ± 12.43% of T-cells, 11.65 ± 6.71% of B-cells, 12.77 ± 7.64% of NK cells, and 0.64 ± 0.35 of NKT cells. Analysis of T-cells subpopulation in the control group showed 38.72 ± 10.96% of CD4+ cells, 29.74 ± 6.65% of CD8+ cells (CD4/CD8 index—1.42) and 12.96 ± 8.31% of activated T-cells (CD3+ HLA-DR). There were no significant changes in all subpopulations of lymphocytes between the control and experimental group (day 0). Desensitization did not affect the percentage of T-cells (0 week—66.11 ± 12.51%; 2 weeks—64.32 ± 10.40%; 6 weeks—64.44 ± 13.62%, 24 weeks—61.35 ± 11.33%), B-cells (0 weeks—11.57 ± 4.66%; 2 weeks—11.11 ± 3.94%; 6 weeks—10.95 ± 4.63%; 24 weeks—13.29 ± 5.96%), NKT-cells (0 week—9.79 ± 7.36%, 2 weeks—12.18 ± 11.17; 6 weeks—11.12 ± 11.03%; 24 weeks—9.69 ± 8.17) and CD8+ T-cells (0 week—30.00 ± 10.78; 2 weeks—30.97 ± 10.92; 6 weeks—29.67 ± 9.95; 24 weeks—31.77 ± 11.58) 2, 6, and 24 weeks after the initiation phase of VIT. However, we observed a significant reduction of CD4+ T-cells (about 20%, p = 0.008, 32.87 ± 12.95%) and activated T-cells (about 26%, p = 0.032, 10.42 ± 8.63%) 24 weeks after the initiation phase of VIT. Interestingly, the results of NKT cells were characterized by the highest viariability in both, study and control group. The results are presented in Fig. 3.

### Regulatory T cells

**Figure 4:**
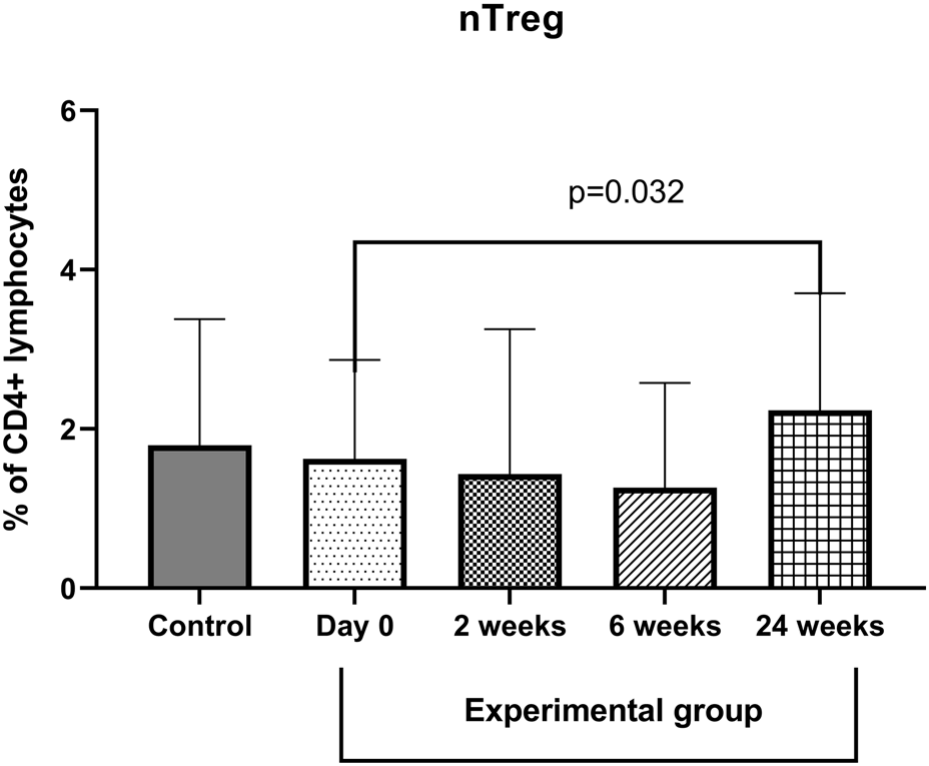
The effect of desensitization with Hymenoptera venom on the nTreg percentage in the blood. Blood was collected at day 0 (control and examined group), and 2, 6, and 24 weeks after the initiation phase of VIT (experimental group only). Results are presented as mean percentage ± SD. *p* level of difference.

The percentage of nTreg in the control group amounted to 1.79 ± 1.59% of CD4 positive lymphocytes. There were no differences between the control and experimental group in nTreg percentage. We did not observe significant changes in the short-time period (2–6 weeks) weeks after the initiation phase of VIT, however, there was a significant increase in the percentage of nTreg in the blood 24 weeks after the initiation phase of VIT (about 37%, p = 0.032, 2.23 ± 1.44%). The results are presented in Fig. 4.

### Complement

**Figure 5:**
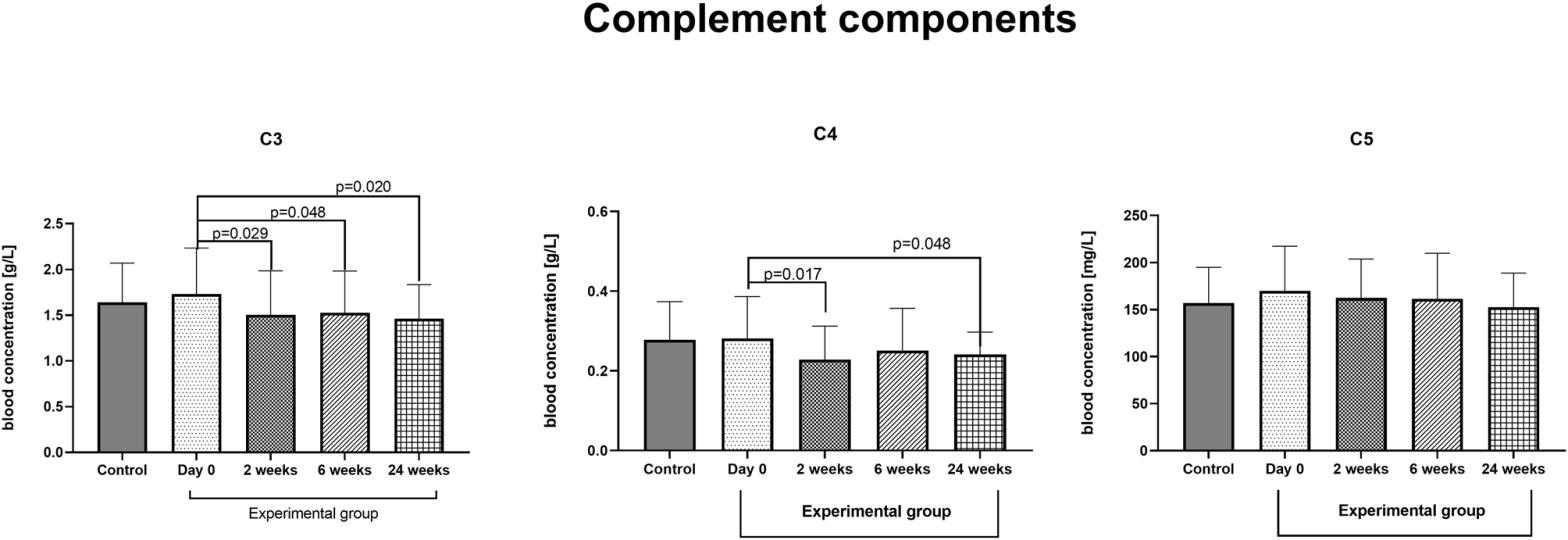
The effect of desensitization with hymenoptera venom on the concentration of selected complement components (C3, C4, and C5) in the blood. Blood was collected at day 0 (control and experimental group), and 2, 6, and 24 weeks after the initiation phase of VIT (examined group only). Results are presented as mean percentage ± SD. *p* level of difference.

The concentration of selected complement components: C3, C4, and C5 in the blood of the control group patients amounted to: 1.64 ± 0.43, 0.28 ± 0.09 g/l, and 157.1 ± 37.78 mg/l. There was no change in the concentration of C3 (1.73 ± 0.50 g/l), C4 (0.28 ± 0.10 g/l), and C5 (169.7 ± 46.8 g/l) components between the control and experimental group (day 0). Desensitization caused a persistent decrease in the concentration of the C3 component [about 15% at 2, 6, and 24 weeks after the initiation phase of VIT, respectively: 1.50 ± 0.47, 1.53 ± 0.45, 1.46 ± 0.37 g/l)] and C4 concentration (2 and 24 weeks after the initiation phase of VIT, about 20%—0.23 ± 0.08 g/l and 15%—0.24 ± 0.05 g/l, respectively). There was no effect of desensitization on the concentration of the C5 complement component (2 weeks—162.3 ± 40.76, 6 weeks—161.59 ± 47.62, 24 weeks—152.52 ± 35.66 g/l). The results are presented in Fig. 5.

### Histamine and tryptase

**Figure 6:**
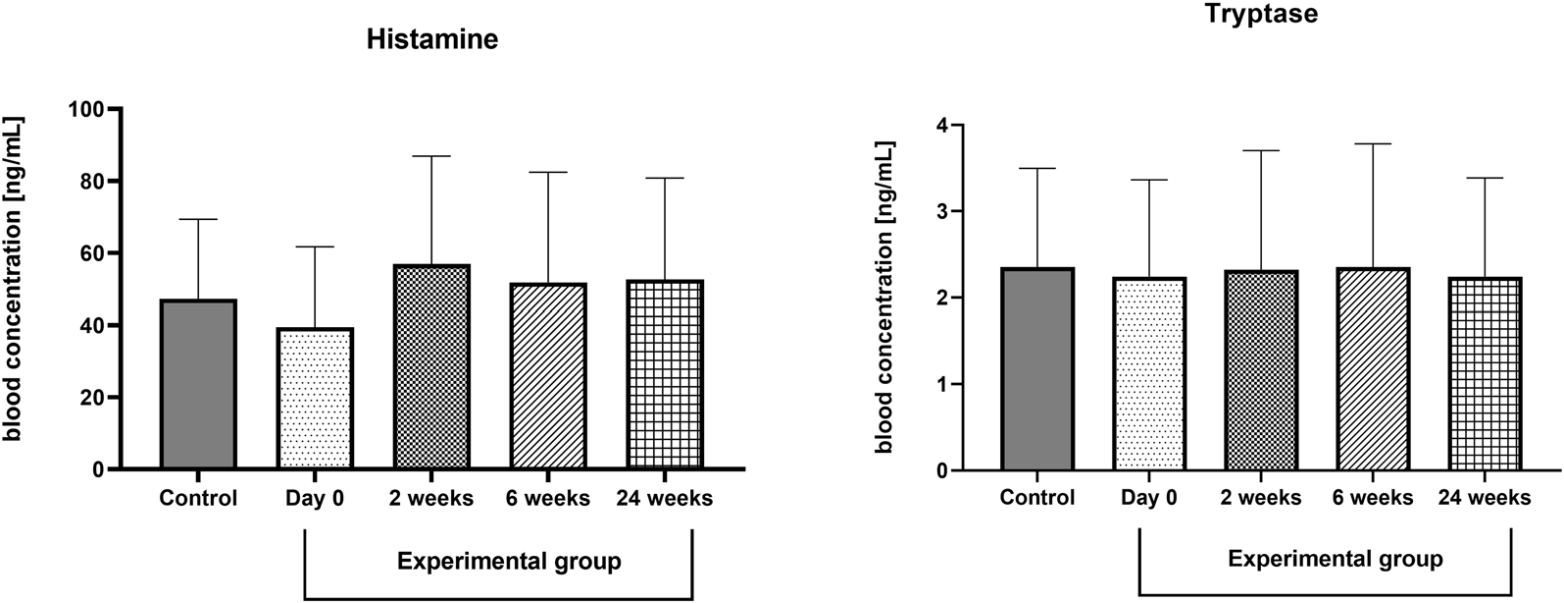
The effect of desensitization with Hymenoptera venom on the concentration of histamine and tryptase in the blood. Blood was collected at day 0 (control and experimental group), and 2, 6, and 24 weeks after the initiation phase of VIT (experimental group only). Results are presented as mean percentage ± SD. *p* level of significance.

The blood concentration of histamine was similar in the control and experimental group at the beginning of the experiment. We did not observe any significant effect at 2, 6, and 24 weeks after the initiation phase of VIT. Similar to histamine, tryptase concentration was unaffected in the exposure group (day 0) and after 2, 6 and 24 weeks after the initiation phase of VIT. The results are presented in Fig. 6.

## Discussion

Allergies are one of the fastest-growing chronic diseases in the world. It is estimated that about 1 in 5 adults suffer from allergies^8,9^. From a clinical point of view, allergy to insect venom constitutes an vital problem mainly due to their turbulent course. An insect sting in sensitized patients initiates a rapid release of inflammatory mediators from mast cells, which causes a strong systemic inflammatory reaction, leading to severe health consequences, including anaphylactic shock and/or death. The rate of systemic sting reactions in epidemiological studies in Europe ranged between 0.3 and 7.5% in adults and 0.15–3.4% in children^10^ and 0.09–0.74 deaths per million inhabitants per year^11,12^. These reactions are classified according to their clinical spectrum and severity, although only 'Müller's classification is exclusively proposed for systemic reactions to HVA^13^. The severity classification is based on a 4-point scale, covering the main symptoms from the mildest (I—urticaria; II—abdominal pain, vomiting); to the most severe, sometimes life-threatening: (III—respiratory disorders; and IV—cardiovascular system: are termed anaphylactic shock). One of the best methods of preventing complications from insect stings is venom insect immunotherapy (VIT). The occurrence of the HVA III–IV reaction is an absolute indication for VIT. In our Clinic at the Military Institute of Medicine in Warsaw, more than 100 patients per year have venom immunotherapy, with average of 3.5 years of VIT treatment.

The main mechanism of VIT treatment is the development of peripheral tolerance. It is mediated by the synthesis and secretion of blocking antibodies, especially IgG4 class, which may inhibit IgE-dependent reactions. This therapy contributes to significant attenuation of anaphylaxis reaction after insect sting for 80–95% of patients^14^. The effect of immunotherapy treatment is associated with basophils and mast cell function. Plewako et al. observed a significant decrease of basophils number in the blood of patients and reduced function of basophils (release of histamine as well as IL-4 and IL-13) within three days after rush immunotherapy^15^. Interestingly, observed changes returned to baseline values 1 week after initiation of VIT treatment. The treatment also caused an increase of CD63+ basophils in the blood (within 1 week), indicating a partial basophils degranulation^16^. Here we did not observe significant changes in the percentage of basophils in peripheral blood however, the first follow-up was performed 2 weeks after initiation of the VIT therefore, the obtained results are consistent. VIT also induces changes in other populations of white blood cells in peripheral blood. Schuerwerght et al.^17^, during a study involving 20 patients with wasp VIT (semirush protocol), observed a significant increase of neutrophils and a decrease of lymphocytes five days after the initiation phase. Our results confirm these observations however, we also show the comparison to the control group. At the beginning of the treatment, the experimental group (III/IV Müller's grade) was characterized by increased percentage of lymphocytes and decreased percentage of neutrophils when compared with the control group (I/II Müller's grade). During the 24 weeks after initation of VIT, the percentage of lymphocytes decreased while the percentage of neutrophils increased to match the values observed in the control group.

Lymphocytes (T and B cells) are the central populations of immune cells responsible for the adaptive response.. Interaction of Th and B-cells, whit appropriate cytokines stimulation, is necessary for the production of antibodies. IFN-γ induces a shift towards Th1 cell and IgG antibody production, while IL-4 favorises the Th2 cells and IgE production^18^. In allergies, the second pathway is dominant. Among the evaluated subpopulations, we observed an increase in the percentage of NK cells as well as a decrease of Th-cells (CD4+) and activated T-cells (HLA-DR+) over the time of the treatment. Our results are in line with the findings of Schuerwerght et al.^17^ even though they did not observe significant changes in the percentage of CD3+, CD4+, CD8+ and CD19+ cells five days or six months after the initial phase of VIT. Instead, the authors reported a significant reduction of IL-4 producing cells in both CD4+ and C8+ subpopulations. Also, other authors showed that VIT decreases IL-4 and IL-13 (Th2 cytokines) and increases INF-γ (Th-1) 15 and 45 days after first-time treatment^19^. These indicated that functional but not quantitative changes of CD4 and CD8 positive cells are responsible for the initiation of immunotolerance on wasp venom. Moreover, the reduction of activated T-cells observed 24 weeks after the initiation phase of VIT treatment suggests a decreased activation of an immune response, probably associated with increased immunotolerance.

There is still not enough data to pinpoint the exact time after initiation of VIT when immunological tolerance is achieved. Zakrzewski et al.^20^ showed no significant changes in IL-10, IL-21, and TGF-β1 2.5- or 24-h after the initiation phase of VIT. Bussmann et al.^21^, in the study investigating early markers of rush immunotherapy, found a significant decrease of tryptophan (induction of tolerance), increased inhibitory receptors for monocytes (ILT3 and ILT4), and elevation of immunosuppressive IL-10 concentration in the blood within the first 5 days. Moreover effect of VIT is prolongated in time. Immunomdulatory cytokines (IL-10, IL-21 and TGF-β1) are mainly produced by regulatory T- and B- regulatory cells. These populations of immune cells are responsible for attenuation of immune response and may occur as natural (n) or induced (i) regulator cells. In the present paper, we assessed the percentage of nTreg cells in the peripheral blood. The percentage of nTreg was increased after VIT treatment which was especially seen 24 weeks post-treatment. This is similar to other authors, which described the elevated level of natural and induced Treg cells in peripheral blood after VIT treatment^21–24^. The question arises when an organism enables the increase of nTreg cells production. In most of the VIT studies, CD25+ or CD25high with FoxP3+ cells are evaluated. These markers do not distinguish nTreg from induced Treg cells. We theorize that at the beginning of VIT iTreg cells are produced and then about 6–8 weeks after the initiation phase of VIT number of the nTreg cells is growing. Whether which cells iTregs o nTreg are dominant, the elevation of those cells in the blood is one of the known mechanism by which VIT treatment affect the immune system response. An increased level of nTreg in the peripheral blood should weaken the immune system's hypersensitivity. This is evidenced by the data from the other studies, in which allergy groups had lower levels of nTregs compared to the healthy volunteers, as well as by our previous studies^25,26^.

Differences among cell subpopulations during VIT, observed by various research groups, are not always homogenous. This is mainly due to the fact that the human population is characterized by high variability of immune cells in the peripheral blood (standards for healthy people are broad i.e., lymphocytes 20–40%). For this reason, observation of changes in the blood might be challenging, and therefore significant differences observed during this study must be especially pronounced.

The complement system is one of the components of the innate immune system, which may be activated by classic pathway (antibody-dependent), lectin pathway (pathogen recognition receptor-dependent), or alternative pathway (independent)^27^. These three independent pathways gather at one common point where the C3 component its cleaves into C3a and C3b by the C3 convertase (classical and lectin pathway) or by spontaneous C3 hydrolysis (alternative pathway). The next step is the cleavage of the C5 component into C5a and C5b, and C5b is the main factor necessary to develop a Membrane Attack Complex (MAC)^28^. Activation of the complement system and causing the main effect also produces anaphylatoxins: C4a, C3a, and C5a. The activity of these complement components varies—the shortest half-life is shown by factors C4a and C3a, and the highest biological activity is shown by anaphylatoxins C5a^29^. Complement factor C3a and C5a exhibited similar functional profiles: induce a local inflammatory response by degranulation of granulocytes, mast cells, and endothelial cells, increase blood permeability, smooth cells contraction, and homing of cells with regenerative properties.

In contrast, C4a inflammation properties are less known and nowadays questionable^30^. Control of the complement system is complicated and precise. One of the possibilities for modulation of the complement system is modulation of the availability of key factors^31^. The role of the C3,C4 and C5 complement components and its anaphylatoxins C3a, C4a, and C5a in the development and promotion of allergies are widely discussed in the literature. It was postulated that C3 and especially C5 with their anaphylatoxins promote the development of Th2 immunity during allergen sensitization, which contributes to asthma^32^. Furthermore, blocking C5 by antibody reduces type 2 responses in a house dust mite-induced murine asthma model^33^. The C3 and C5 components' significant role was also confirmed in allergic nasal mucosa^34^ and atopic dermatitis^35^. C3a receptor antagonism is considered a therapeutic target for chronic rhinosinusitis^36^. Therefore in the present work, we evaluated the effect of desensitization on the concentration of C3, C4, and C5 components. Few papers describe the effect of Hymenoptera venom and desensitization on complement factors. It is known that Hymenoptera venom may activate the complement system's alternative pathway by increasing the cleavage of the C3 component^37^. The cleavage of C3 is more severe when C3 concentration is higher^38^. Here we observed a significant reduction in the blood concentration of C3 and C4 components, especially in the long period after desensitization with Hymenoptera venom, without any changes of the C5 concentration. These results are in agreement with Matysiak et al.^39^, who showed decreased levels of C3 and C4 in serum of patients after venom immunotherapy (VIT) measured by MALDI-TOF MS technique. This may be a new mechanism by which desensitization modulates the immune 'system's response to Hymenoptera venom.

Histamine is one of the main inflammatory compounds released rapidly after bounding of antigen to specific IgE antibodies of the surface form mast cells. Histamine plays a significant role in the pathogenesis of allergic disease mainly by regulating T cells differentiation from Th0 to Th2 cells^40^. Moreover, during VIT, there was observed a decrease in basophils numbers and produced mediators can be observed as presented by Novac and Maintz^41,42^. It is also known that histamine exerts its biological effects through receptors. Among four different histamine receptors, type HR2 plays an important role in peripheral antigen tolerance. HR 2 is mainly involved in tolerogenic immune response^43^ Histamine via HR2 induces IL-10 production by antigen producing cells, increases the suppressive effect of TGF-B on T cella, and decreases IL-5 and IL-13 production through the Th2 Lymphocytes. Venom immunotherapy caused a decrease of HRs^44^, besides the HR2, which is dominant in allergen-specific T cells venom immunotherapy^45^. In the study performed by Pierkers et al.^46^ VIT (rush therapy reaching a maintenance dose of 100 μg venom injected subcutaneously within 1 week) caused a significant reduction of histamine release from heparinized blood after wasp venom treatment. Also, in basophils isolated from 'children's blood, allergen-specific conventional immunotherapy caused a significant time-dependent decrease of histamine release after *Dermatophagoides farina* stimulation^47^. In both works, however, the measurement of histamine concentration was performed in isolated blood after antigen stimulation. Here, we focused on blood histamine concentrations in patients undergoing venom immunotherapy. We did not observe any significant changes in histamine concentration in the blood of the control and experimental group in the studied time-points. Histamine is released rapidly, and it appears in the blood within 2.30 min and returns to baseline concentration after 30 min^48^. It may be why there were no significant changes in the concentrations of histamine in the blood was found in our research. Moreover, histamine production and release in allergy are associated with basophils, which decreased after VIT only in a short-perspective (3 days) T^49^. Interestingly, the authors of this paper observed a decrease in the basophil intracellular histamine content, which was not studied in the present study. We did not observe any changes in the concentration of tryptase—the main enzyme marker of mast cell burden and mast cell function. The concertation of the enzyme is generally higher in patients with higher grades of the Müller scale^50^. In children suffering from wasp or honeybee sting allergy, rush VIT protocol resulted in increase tryptase 8 days after first dose. On the other hand long term evaluation of VIT revealed a 2.5% per year decrease of serum tryptase concentration in adult patients (about 2.5% per year)^51^. In the present study, wefocused on the adult patients that were observed for up to 24 weeks, therefore some short-term and long-term effects of venom immunotherapy were not seen.

## Conclusions

There is plenty of data available on short-term modifications of immune system components after VIT treatment. However, our knowledge about the long-term effects of VIT treatments is still insufficient. Here, we evaluated the long-term impact of VIT on patients with previous systemic anaphylactic reactions ('Müller's grade reactions III and IV) after a wasp sting. We observed an increase of natural Treg cells in the peripheral blood of those patients, and we found that C3 and C4 complement components in the blood are decreased. Those changes were time-dependent, with the highest differences observed 24 weeks after VIT treatment.

## Materials and methods

### Patients

The study includes patients from the Department of Infectious Diseases and Allergology, Military Institute of Medicine (Warsaw, Poland) who reported an overreaction of the organism after contact with Hymenoptera venom. The study included 61 patients—18 in the control group and 43 in the experimental group. The inclusion criterion was the diagnosis of allergy to wasp venom and previous systemic anaphylactic reaction. 'Müller's grade reactions III and IV were treated as the study group, while 'Müller's grades I and II were considered the control group. The 'study's general exclusion criteria included cardiovascular and oncological diseases and medication with systemic drugs that reduced immune system functions.

Additionally, each 'patient's level of IgE and specific IgE (sIgE) against wasp, bee and hornet was checked. The level of sIgE above 1 was exclusion criteria from the control group and below 2 (wasp sIgE) was exclusion criteria from the experimental group (Fig. 1). There were 8 women and 10 men in the control group, and the average group age was 50.7 ± 13.6 years. There were 22 women and 21 men in the experimental group with an average age of 49.6 ± 12.5 years. Clinical data of patients from control and experimental groups are presented in Table 1.

**Table 1:** Clinical data of patients from control and experimental groups.

No.	Age	Sex	Weight	Non-wasp allergies	The time point of the last sting
**Control group**					
1	66	M	89	n/a	2017
2	20	F	74	n/a	2018
3	53	F	86	Food allergy	2017
4	42	M	63	n/a	2017
5	60	M	90	n/a	2017
6	66	F	70	n/a	2018
7	39	F	50	Pollen allergy	2018
8	50	M	96	n/a	2018
9	49	M	97	n/a	2017
10	44	M	93	n/a	2017
11	73	M	85	n/a	2018
12	52	M	98	n/a	2017
13	16	M	67	n/a	2018
14	29	M	75	Avena	2017
15	71	F	72	n/a	2017
16	45	F	73	n/a	2018
17	68	F	78	n/a	2017
18	56	M	80	n/a	2017
**Experimental group**					
1	35	F	60	n/a	2018
2	45	F	86	Drugs (Penicillium, Metronidazole)	2018
3	50	M	102	n/a	2017
4	42	F	60	n/a	2018
5	27	M	100	n/a	2017
6	58	F	104	n/a	2018
7	43	M	80	n/a	2018
8	30	M	65	n/a	2018
9	38	M	94	n/a	2017
10	44	F	72	Pollen allergy	2018
11	43	F	60	n/a	2017
13	42	M	97	n/a	2018
14	63	F	74	n/a	2018
15	34	M	80	n/a	2017
16	47	M	77	n/a	2017
17	50	M	98	n/a	2018
18	67	F	50	n/a	2018
19	50	M	73	n/a	2017
20	47	M	95	Aspirin	2018
21	58	M	97	n/a	2018
22	67	F	58	n/a	2018
23	55	F	58	Pollen allergy	2017
24	44	M	95	n/a	2017
25	50	M	110	n/a	2017
26	62	M	105	n/a	2017
27	36	M	82	Pollen allergy	2018
28	63	F	66	n/a	2017
29	53	F	100	Drugs (metamizole, penicillium)	2017
30	51	F	79	Drug (piroxicam)	2017
32	27	F	54	n/a	2019
33	24	F	59	n/a	2017
34	67	F	58	n/a	2018
35	48	F	68	n/a	2017
36	39	F	58	Pollen allergy	2018
37	46	F	94	Pollen allergy	2019
38	72	M	105	n/a	2018
39	37	F	58	n/a	2017
41	38	F	50	n/a	2018
42	41	M	80	n/a	2018
43	60	M	112	n/a	2018

The study obtained a positive opinion of the Bioethics Committee of the Military Institute of Medicine in Warsaw (Resolution No. 130/WIM/2018). All patients signed an informed consent form, and those who were undergoing venom immunotherapy received oral antihistamines as a pretreatment before the ultra-rush induction phase and during the maintenance treatment.

### IgE analysis

Peripheral blood samples were collected by the "clot" method. After 20 min blood was centrifuged (20 min, 2000×*g*), and then serum was collected. Total IgE and specific (s) IgE concentrations were measured in the serum samples by a fluorometric method using UniCAP machine (Pharmacia, HVD Holding AG Sp. z o.o). Anti-IgE, covalently coupled to the reaction vessel, reacted with the sample's total IgE. After washing, enzyme-labeled antibodies against IgE were added to form a complex. After incubation, unbound enzyme-anti-IgE was washed away, and the bound complex was then incubated with a developing agent. After stopping the reaction, the fluorescence of the eluate was measured. To evaluate the results, the response of patient 'samples' was compared directly to the response of the calibrators.

### Desensitization

In the examined group of subjects, an ultra-rush protocol was used at the induction phase of VIT. The wasp venom (Venomenhal^®^, 120 µg of wasp venom/vial) was administered subcutaneously only in the examined group with the increasing doses of 0.1 μg, 1 μg, 10 μg, 20 μg, 30 μg, and finally 40 μg in the 30 min intervals (101.1 μg in total, induction phase). After the induction phase, patients received a maintenance dose: 100 μg (40 μg as an initial dose and 60 μg after 30 min) after 2 weeks and 100 μg of a vaccine every 4 weeks of the maintenance period for about 3.5 ± 0.5 years. Summarized protocols are presented in Table 2.

**Table 2:** Protocol of rush venom immunotherapy,

Weeks after the initiation phase of VIT	Venom extract concentration, μg/mL	Cumulative doses, μg/mL
0	0.1 + 1 + 10 + 20 + 30 + 40	100.1
2	40 + 60	200.1
6	100	300.1
10	100	400.1
14	100	500.1
18	100	600.1
22	100	700.1
26	100	800.1
30	100	900.1

and then 100 μg every four weeks for about 3.5 ± 0.5 years.

### Blood collection

Twenty ml of peripheral vein blood were drawn to EDTA tube or the tube for serum preparation immediately before the start of ultra-rush and before subcutaneously vaccine injection (2, 6, and 24 weeks after the induction phase). After collection, blood was immediately transported to the laboratory.

### Percentage analysis of WBC and lymphocytes

Analysis of WBC and Lymphocytes phenotypes was performed as previously described^52^. Determination of white blood cells phenotypes was identified by morphological parameters (FSC/SSC) and tube A (CD45+/CD14+) from Simultest™—IMK Plus Kit; (BD Biosciences, Poland). Blood lymphocyte immunophenotype was determined by BD Simultest™—IMK Plus Kit (BD Biosciences, Poland). Briefly, after collection of peripheral blood, samples (100 µL) were incubated 20 min with appropriate antibodies: Leucogate™ (A CD45 FITC/CD14 PE), Isotype Control (IgG1 FITC/IgG2a PE), CD3 FITC/CD19 PE, CD3 FITC/CD4 PE, CD3 FITC/CD8 PE, and CD3 FITC/CD16 PE + CD56 PE. Then erythrocytes lysis was performed (BD FACS Lysing Solution, BD Biosciences, Poland)—10 min at room temperature (RT) in the dark. After lysis, cells were washed twice with 2 ml of phosphate-buffered saline (PBS) and fixed in 200 μl 1% paraformaldehyde (PFA) in PBS and acquired in flow cytometry (Facs Calibur, BD Warsaw). Percentage distribution of white blood cells (lymphocytes, monocytes, granulocytes, neutrophils, eosinophils) was done using CD45 FITC, CD14 PE antibody, and FSC/SSC determination. The lymphocyte phenotypes are presented as the mean percentage of lymphocytes ± SD.

### nTreg cells analysis

Analysis of nTreg cells was performed as previously described^52^. Briefly, blood samples (100 µl) were stained with primary antibodies CD4-PerCP, CD25-APC, CD127-FITC (extracellular staining, BD Bioscence, Poland), or appropriate isotype control with additionally CD4-PerCP antibody. Blood samples were incubated 20 min at room temperature in the dark, and then erythrocytes lysis was performed. After double PBS wash, cells were fixed and permeabilized in Fixation/Permeabilization buffer (BD Pharmingen, Poland) and stained with FoxP3 PE or isotype IgG1 kappa PE antibody (45 min at room temperature in the dark). Afterward, cells were washed twice with PBS, fixed in 300 μl of 1% PFA in PBS solution, and analyzed by flow cytometry. The acquisition was stopped after 10,000 counts of CD4 PerCP positive cells. We defined natural Treg (nTreg) cells as: CD4^+^/CD25^high^/CD127^low^/FoxP3^+^.

### Complement system analysis

Evaluation of serum complement C3 and C4 components were performed by the nephelometric method using MininephTM C3 or C4 Kit reagents from Binding Site Group Ltd. (Birmingham, UK). The kits were designed for the in vitro measurement of human C3 and C4 in serum using the MININEPHPLUS analyzer. We used the recommended dilution for C3 and C4 analyses (1/5). Assays were performed according to the 'manufacturer's instructions.

The concentration of the C5 complement was determined by the radial immunodiffusion method using the Human Complement C5 BINDARID Radial Immunodiffusion Kit (Binding Site Group Ltd., Birmingham, UK). C5 serum concentration was analyzed using the calibration curve (3 calibrators included in the kit at the concentrations of 20 mg/l, 120 mg/l, 200 mg/l). The test was performed according to the 'manufacturer's instructions. Measurement of the precipitin ring diameter in the calibrations and test samples was performed after 72 h.

### Histamine and tryptase

According to the 'manufacturer's recommendations, serum histamine and tryptase concentrations were evaluated using the ELISA method (FineTest^®^, Wuhan, China). 50 μl of serum was used for histamine and 100 μl of serum for tryptase assessment. All samples were evaluated in duplicate. Results are presented as the mean ± SD.

### Statistical analysis

All results were presented as the mean ± standard deviation (SD). The data distribution was evaluated using the Shapiro–Wilk test. For normally distributed data One-way ANOVA with Bonferroni correction and T-test were used. The rest of the data was evaluated using non-parametric One-way ANOVA with Kruskal–Wallis correction and Mann–Whitney Test. GraphPad Prism software (version 9.2.0; GraphPad Software, Inc., La Jolla, CA, USA) was used for all evaluations. p < 0.05 was considered statistically significant.

### Institutional review board statement

The study was conducted according to the guidelines of the Declaration of Helsinki, and approved by Ethics Committee of Military Institute of Medicine, Warsaw Poland, resolution No. 130/WIM/2018.

## References

[1] Cichocka-Jarosz E (2019). Hymenoptera sting in the head and neck region is not a risk factor for grade IV systemic reactions in patients with venom allergy. Pol. Arch. Intern. Med..

[2] Pérez Pimiento AJ (2007). Systemic reactions to wasp sting: Is the clinical pattern related to age, sex and atopy?. Allergol. Immunopathol. (Madr).

[3] Schiener M, Graessel A, Ollert M, Schmidt-Weber CB, Blank S (2017). Allergen-specific immunotherapy of Hymenoptera venom allergy—Also a matter of diagnosis. Hum. Vaccin. Immunother..

[4] Akdis CA, Akdis M (2015). Mechanisms of allergen-specific immunotherapy and immune tolerance to allergens. World Allergy Organ J.

[5] Ozdemir C, Kucuksezer UC, Akdis M, Akdis CA (2011). Mechanisms of immunotherapy to wasp and bee venom. Clin. Exp. Allergy.

[6] Workman CJ, Szymczak-Workman AL, Collison LW, Pillai MR, Vignali DAA (2009). The development and function of regulatory T cells. Cell Mol. Life Sci..

[7] Müller U, Helbling A, Berchtold E (1992). Immunotherapy with honeybee venom and yellow jacket venom is different regarding efficacy and safety. J. Allergy Clin. Immunol..

[8] Seité S, Taieb C, Pham-Thi N, Barbaud A (2021). Allergy prevalence in france and skin impact—Epidemiological survey of a representative sample of French adults. Clin. Cosmet. Investig. Dermatol..

[9] Nachshon L (2021). Characteristics and associated morbidities of young adults with misconceived food allergy: A cross-sectional study. EClinicalMedicine.

[10] Bilò BM, Bonifazi F (2008). Epidemiology of insect-venom anaphylaxis. Curr. Opin. Allergy Clin. Immunol..

[11] Prado M, Quirós D, Lomonte B (2009). Mortality due to Hymenoptera stings in Costa Rica, 1985–2006. Rev. Panam. Salud Publica.

[12] Turner PJ (2015). Increase in anaphylaxis-related hospitalizations but no increase in fatalities: An analysis of United Kingdom national anaphylaxis data, 1992–2012. J. Allergy Clin. Immunol..

[13] Mueller, U. R. & Mieller. *Insect Sting Allergy, Clinical Picture, Diagnosis, and Treatment*. (1990).

[14] Sturm GJ (2018). EAACI guidelines on allergen immunotherapy: Hymenoptera venom allergy. Allergy.

[15] Plewako H (2006). Basophil interleukin 4 and interleukin 13 production is suppressed during the early phase of rush immunotherapy. Int. Arch. Allergy Immunol..

[16] Siegmund R, Vogelsang H, Machnik A, Herrmann D (2000). Surface membrane antigen alteration on blood basophils in patients with Hymenoptera venom allergy under immunotherapy. J. Allergy Clin. Immunol..

[17] Schuerwegh AJ, De Clerck LS, Bridts CH, Stevens WJ (2001). Wasp venom immunotherapy induces a shift from IL-4-producing towards interferon-gamma-producing CD4+ and CD8+ T lymphocytes. Clin. Exp. Allergy.

[18] Paludan SR (1998). Interleukin-4 and interferon-gamma: The quintessence of a mutual antagonistic relationship. Scand. J. Immunol..

[19] Mamessier E, Birnbaum J, Dupuy P, Vervloet D, Magnan A (2006). Ultra-rush venom immunotherapy induces differential T cell activation and regulatory patterns according to the severity of allergy. Clin. Exp. Allergy.

[20] Zakrzewski A (2019). How fast does wasp venom immunotherapy affect a regulatory T cell subpopulation (CD4+ CD25+ Foxp3+) and the synthesis of interleukins 10, 21 and transforming growth factor β1?. Postepy Dermatol. Alergol..

[21] Bussmann C (2010). Early markers for protective mechanisms during rush venom immunotherapy. Allergy.

[22] Bohle B (2007). Sublingual immunotherapy induces IL-10-producing T regulatory cells, allergen-specific T-cell tolerance, and immune deviation. J. Allergy Clin. Immunol..

[23] Caramalho I (2015). Bee venom enhances the differentiation of human regulatory T cells. Allergy.

[24] Demšar Luzar A, Korošec P, Košnik M, Zidarn M, Rijavec M (2021). Hymenoptera venom immunotherapy: Immune mechanisms of induced protection and tolerance. Cells.

[25] Lipińska-Opałka A, Wawrzyniak A, Lewicki S, Zdanowski R, Kalicki B (2017). Evaluation of immune indices and serum vitamin D content in children with atopic dermatitis. Adv. Exp. Med. Biol..

[26] Wawrzyniak A (2017). Evaluation of selected immunological parameters and the concentration of vitamin D in children with asthma. Case-control study. Cent. Eur. J. Immunol..

[27] Dunkelberger JR, Song W-C (2010). Complement and its role in innate and adaptive immune responses. Cell Res..

[28] Serna M, Giles JL, Morgan BP, Bubeck D (2016). Structural basis of complement membrane attack complex formation. Nat. Commun..

[29] Reber LL, Hernandez JD, Galli SJ (2017). The pathophysiology of anaphylaxis. J. Allergy Clin. Immunol..

[30] Barnum SR (2015). C4a: An anaphylatoxin in name only. J Innate Immun..

[31] Ricklin D, Reis ES, Mastellos DC, Gros P, Lambris JD (2016). Complement component C3—The “Swiss Army Knife” of innate immunity and host defense. Immunol. Rev..

[32] Zhang X, Köhl J (2010). A complex role for complement in allergic asthma. Expert Rev. Clin. Immunol..

[33] Yang J (2019). Complement factor C5 inhibition reduces type 2 responses without affecting group 2 innate lymphoid cells in a house dust mite induced murine asthma model. Respir. Res..

[34] Mezei G, Varga L, Veres A, Füst G, Cserháti E (2001). Complement activation in the nasal mucosa following nasal ragweed-allergen challenge. Pediatr. Allergy Immunol.

[35] Kapp A, Schöpf E (1985). Involvement of complement in atopic dermatitis. Acta Derm. Venereol. Suppl. (Stockh).

[36] Mulligan JK (2018). C3a receptor antagonism as a novel therapeutic target for chronic rhinosinusitis. Mucosal Immunol..

[37] De Carolis C, Perricone R, De Sanctis G, Fontana L (1982). Complement activation by hymenoptera venom allergenic extracts. J. Allergy Clin. Immunol..

[38] Liu Z (2016). Elevated serum complement factors 3 and 4 are strong inflammatory markers of the metabolic syndrome development: A longitudinal cohort study. Sci. Rep..

[39] Matysiak J (2021). Association between venom immunotherapy and changes in serum protein—Peptide patterns. Vaccines (Basel).

[40] Thangam EB (2018). The role of histamine and histamine receptors in mast cell-mediated allergy and inflammation: The hunt for new therapeutic targets. Front. Immunol..

[41] Novak N (2012). Early suppression of basophil activation during allergen-specific immunotherapy by histamine receptor 2. J. Allergy Clin. Immunol..

[42] Maintz L, Bussmann C, Bieber T, Novak N (2009). Contribution of histamine metabolism to tachyphylaxis during the buildup phase of rush immunotherapy. J. Allergy Clin. Immunol..

[43] Cavkaytar O, Akdis CA, Akdis M (2014). Modulation of immune responses by immunotherapy in allergic diseases. Curr. Opin. Pharmacol..

[44] Jutel, M., Żak-Nejmark, T., Wrzyszcz, M. & Małolepszy, J. Ultra-rush bee venom immunotherapy results in decrease of histamine receptor expression on peripheral blood CD4 + lymphocytes. *J. Allergy Clin. Immunol.* **99**, (1997).

[45] Müller UR (2008). Clinical and immunologic effects of H1 antihistamine preventive medication during honeybee venom immunotherapy. J. Allergy Clin. Immunol..

[46] Pierkes M (1999). Decreased release of histamine and sulfidoleukotrienes by human peripheral blood leukocytes after wasp venom immunotherapy is partially due to induction of IL-10 and IFN-γ production of T cells. J. Allergy Clin. Immunol..

[47] Shim J-Y, Kim B-S, Cho S-H, Min K-U, Hong S-J (2003). Allergen-specific conventional immunotherapy decreases immunoglobulin E-mediated basophil histamine releasability. Clin. Exp. Allergy.

[48] Bachert C (2002). The role of histamine in allergic disease: Re-appraisal of its inflammatory potential. Allergy.

[49] Nullens S (2013). Basophilic histamine content and release during venom immunotherapy: Insights by flow cytometry. Cytometry B Clin. Cytom..

[50] Cichocka-Jarosz E (2011). Serum tryptase level is a better predictor of systemic side effects than prostaglandin D2 metabolites during venom immunotherapy in children. J. Investig. Allergol. Clin. Immunol..

[51] Dugas-Breit S (2010). Serum concentration of baseline mast cell tryptase: Evidence for a decline during long-term immunotherapy for Hymenoptera venom allergy. Clin. Exp. Allergy.

[52] Kalicki B (2013). Examination of correlation between vitamin D-3 (25-OHD3) concentration and percentage of regulatory T lymphocytes (FoxP3) in children with allergy symptoms. Central Eur. J. Immunol..

